# Therapeutic Potential of Polyphenols in Cardiac Fibrosis

**DOI:** 10.3389/fphar.2018.00122

**Published:** 2018-02-15

**Authors:** Ning Zhang, Wen-Ying Wei, Ling-Li Li, Can Hu, Qi-Zhu Tang

**Affiliations:** ^1^Department of Cardiology, Renmin Hospital of Wuhan University, Wuhan, China; ^2^Cardiovascular Research Institute of Wuhan University, Wuhan, China; ^3^Hubei Key Laboratory of Cardiology, Wuhan, China

**Keywords:** cardiac fibrosis, polyphenols, anti-fibrotic, therapy, signaling pathway

## Abstract

Cardiac fibrosis, in response to injury and stress, is central to a broad constellation of cardiovascular diseases. Fibrosis decreases myocardial wall compliance due to extracellular matrix (ECM) accumulation, leading to impaired systolic and diastolic function and causing arrhythmogenesis. Although some conventional drugs, such as β-blockers and renin-angiotensin-aldosterone system (RAAS) inhibitors, have been shown to alleviate cardiac fibrosis in clinical trials, these traditional therapies do not tend to target all the fibrosis-associated mechanisms, and do not hamper the progression of cardiac fibrosis in patients with heart failure. Polyphenols are present in vegetables, fruits, and beverages and had been proposed as attenuators of cardiac fibrosis in different models of cardiovascular diseases. Together with results found in the literature, we can show that some polyphenols exert anti-fibrotic and myocardial protective effects by mediating inflammation, oxidative stress, and fibrotic molecular signals. This review considers an overview of the mechanisms of cardiac fibrosis, illustrates their involvement in different animal models of cardiac fibrosis treated with some polyphenols and projects the future direction and therapeutic potential of polyphenols on cardiac fibrosis.

## Introduction

Cardiac fibrosis is a hallmark of numerous cardiovascular diseases including hypertension, myocardial infarction (MI), and ischemic, dilated, and hypertrophic cardiomyopathy. Cardiac fibrosis not only interferes with the systolic and diastolic functions and but is also the main determinant of malignant arrhythmias and consequently increases the risk of sudden cardiac death (Roche et al., 2015). There are two patterns of cardiac fibrosis presentation: regional fibrosis and diffuse fibrosis. Regional fibrosis mainly occurs in the healing infarcted ventricle following coronary occlusion, ultimately resulting in the formation of a collagen-based scar (Prabhu and Frangogiannis, 2016). On the other hand, diffuse fibrosis is associated with cardiac remodeling in the conditions of pressure and/or volume overload, metabolic disorder, or ischemic insults and is characterized by unbalanced collagen turnover and excessive diffuse collagen deposition in the interstitial spaces (Frangogiannis, 2017). Some fibrotic factors, such as cytokines, chemokines, growth factors, hormones, and reactive oxygen species (ROS), are responsible for the activation of fibroblasts and the alteration of extracellular matrix (ECM) (Heymans et al., 2015).

A constellation of mechanisms and signaling pathways have been involved in fibroblast activation and pathological remodeling. Mediation of these mechanisms and molecular signals have provoked an intense scientific interest as they work out a novel therapeutic strategy for cardiac fibrosis. At present, the inhibition of cardiac fibrosis and its adverse complications is mainly focused on the conventional drugs, including angiotensin-converting enzyme inhibitors (ACEI), aldosterone antagonists, β-blocker, and statins (Zannad et al., 2000; Bauersachs et al., 2001; Klapholz, 2009; Van de Werf, 2014). These interventions could result in a partial recovery of cardiac function; nevertheless, these effects are secondary to the correction of the underlying cardiac dysfunction mechanisms rather than alleviating fibrosis directly. Moreover, multiple lines of evidence have indicated that pirfenidone alleviates cardiac remodeling in hypertensive rats and pressure-overload in hypertrophic hearts (Mirkovic et al., 2002; Wang et al., 2013). Pirfenidone also decreased infarct scar and improved left ventricle function following cardiac infarction (Nguyen et al., 2010). The other anti-fibrotic drug currently approved for clinical treatment in human idiopathic pulmonary fibrosis (IPF) was nintedanib, just as with pirfenidone (Bando, 2016). Nintedanib is also the subject of ongoing clinical trials for systemic sclerosis. Although nintedanib and pirfenidone show promise for the alleviation of cardiac fibrosis, there are no clinical results to support that these drugs are specifically targeted to the intracellular mechanisms of fibrosis. Therefore, the urgent challenge we are confronting is to find novel potential agents to retard cardiac fibrosis.

Polyphenols are found in plant-derived foods and beverages. Collective evidence has indicated that natural polyphenols possess multiple protective effects against cardiovascular diseases (Raj et al., 2014; Niu et al., 2015). Additionally, polyphenols classified to different subclasses have been reported to alleviate cardiac dysfunction and fibrosis following cardiac injury. In this review, we will examine the role of associated mechanisms in the progression of cardiac fibrosis, illustrate their involvement in the current and emerging intervention of polyphenols, and identify the promising targets of polyphenols for basic and applied cardiac research.

## Molecular mechanism and signals associated with cardiac fibrosis

**Figure 1:**
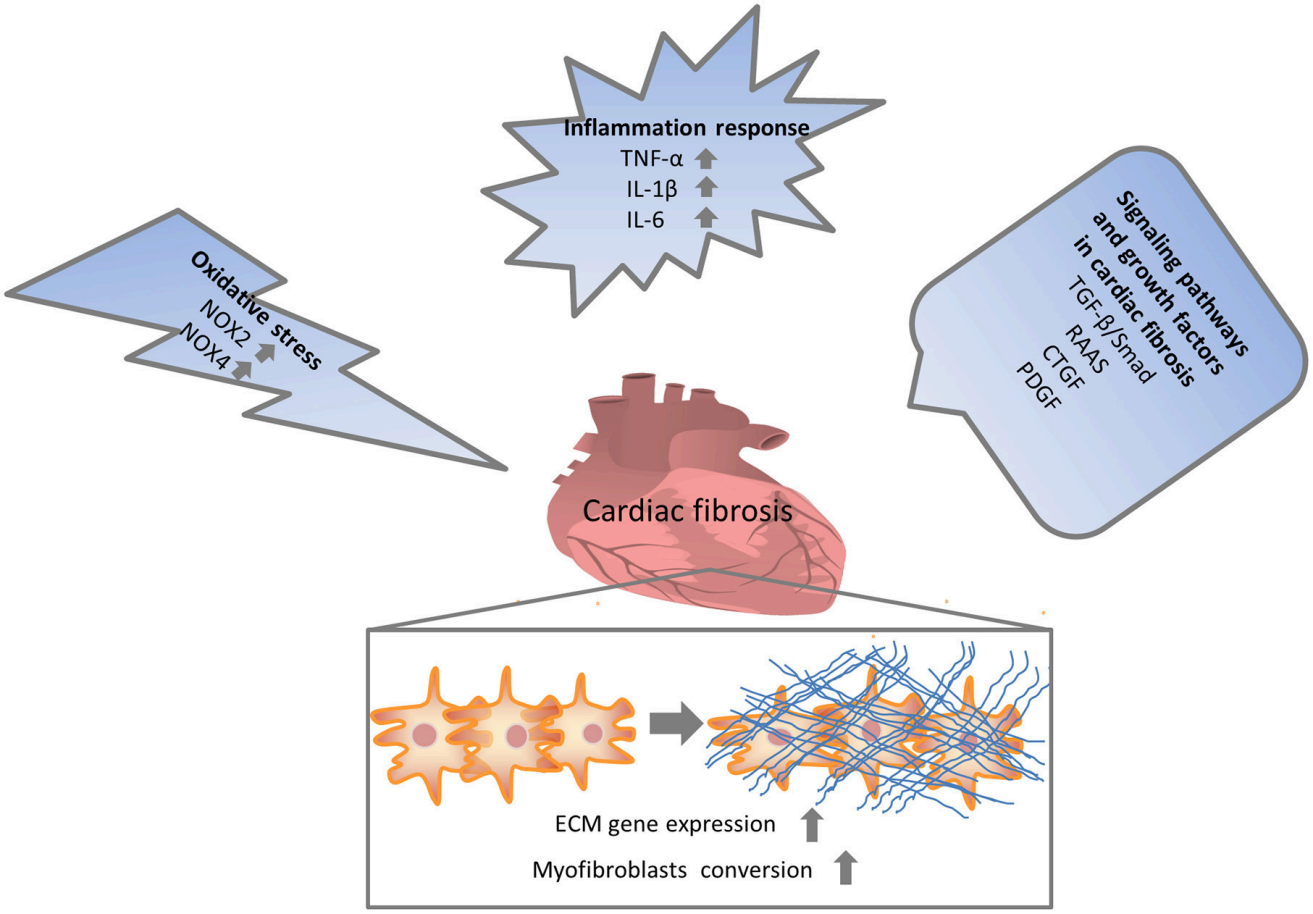
Multiple mechanisms involved in cardiac fibrosis. Enormous initiating insults contributed to oxidative stress, inflammation and increased growth factors, which in turn directly or indirectly enhanced cardiac fibrosis. NOX, NAPDH oxidase; TNF, tumor necrosis factor; IL, interleukin; TGF, transforming growth factor; RAAS, renin-angiotensin-aldosterone system; CTGF, connective tissue growth factor; PDGF, platelet-derived growth factor.

Numerous molecular signals involved in the pathogenesis of cardiac fibrosis, and the identification of these molecular signals implicated with the initiation, progression, and regression of the fibrotic response will help us to effectually target the major culprit to retard cardiac fibrosis (Kong et al., 2014). Inflammatory signals could activate myofibroblasts and seem to play a vital role in regional and diffuse fibrosis. Oxidative stress manifested as an imbalance between a ROS generation and the capacity of anti-oxidant defense systems has been regarded as the most important mechanism in cardiac fibrosis. Other complex cellular mediators, including the transforming growth factor-β (TGF-β), renin-angiotensin-aldosterone system (RAAS), and platelet-derived growth factor (PDGF), also appear to be implicated in cardiac fibrosis. Therefore, intricate cellular and molecular mechanisms contribute to the pathogenesis of cardiac fibrosis (Figure 1).

### Inflammation in cardiac fibrosis

The production of inflammatory signaling molecules during cardiac injury and hypertrophic remodeling can contribute to hypertrophic and fibrotic responses. Activated immune cells, including macrophages, monocytes, lymphocytes, and mast cells, are involved in orchestrating cardiac fibrosis, as reviewed by Ryan A. Frieler (Frieler and Mortensen, 2015). In a mouse model of cardiac infarction, pro-inflammatory cytokines, such as tumor necrosis factor-α, interleukin-1β (IL-1β), and IL-6 released by infiltrated neutrophils and macrophages, could exert a significant role to induce the proliferation of resident fibroblasts and activate myofibroblasts, resulting in cardiac fibrosis (Christia et al., 2013). Moreover, activated cardiac fibroblasts (CFs) also generate numerous cytokines and growth factors that mediate ECM remodeling via autocrine and paracrine mechanisms. After acute cardiac insult, IL-1β enhances fibroblasts migration through the mitogen-activated protein kinase (MAPK) pathway (Mitchell et al., 2007), and IL-6 is known to promote fibroblast proliferation and aggravate myocardial fibrosis (Banerjee et al., 2009). The inflammatory response can also be regulated or induced by cardiac endothelial cells, which recruit macrophages and monocytes through the production of a wide range of cytokines and pro-fibrotic mediators, including TGF-β, fibroblasts growth factor, and endothelin-1 (Wynn, 2008). In humans, an increased level of stressed-induced cytokines, including TNF-α, IL-6, and IL-1β, correlates with a poor prognosis in patients with heart failure (Frieler and Mortensen, 2015). Extensive studies have indicated a significant role of inflammation in the fibrotic response, where the inhibition of inflammatory factors could provide an effective therapeutic strategy in the treatment of cardiac fibrosis.

### Oxidative stress in cardiac fibrosis

Clinical and experimental studies have indicated that oxidative stress, which is defined as the imbalance of excess ROS production and anti-oxidant defense, is enhanced in cardiac remodeling (Wu et al., 2017b). In the cardiac tissues, ROS is mainly sourced from (i) the membrane-bound enzyme complex NADPH oxidase (NOX), (ii) the mitochondrial respiratory chain, and (iii) uncoupled endothelial nitric oxide (NO) synthase (eNOS). Moreover, ROS activates a wide range of hypertrophy signaling kinases and transcription factors and regulates apoptosis. ROS also enhance CF proliferation and stimulate the matrix metalloproteinases (MMPs), resulting in cardiac fibrosis (Tsutsui et al., 2011).

Recent studies demonstrated that NOXs, a family of enzymes implicated in the generation of ROS, are involved in myocardial fibrosis and heart failure progression (Heymans et al., 2015). All forms of NOX, NOX2, and NOX4 are predominantly expressed in the myocardium. In the pathological condition, the expression of NOX increases in the end-stage failing human heart, and cardiac hypertrophy appears as a marked source of increased cardiac ROS. Cardiac hypertrophy induced by Ang II is blunted in gp91^phox^ deficient mice (Shanmugam et al., 2011); however, this protective effect in the deletion of gp91phox subunit in mice is abolished in pressure-overload-induced cardiac hypertrophy (Maytin et al., 2004). In particular, NOX2 enhances myocardial fibrosis stimulated by TGF-β, Ang II stimulation in a rat (Johar et al., 2006; Miguel-Carrasco et al., 2012). Endothelial-specific overexpression of NOX2 promotes interstitial cardiac fibrosis via enhancement of the pro-inflammatory effect and endothelial–mesenchymal transition (Murdoch et al., 2014). Interestingly, eliminating NOX2-induced superoxide anions in CFs retards collagen generation (Lijnen et al., 2012). The previous study also found NOX4 mediates the TGF-β-stimulated conversion of CFs to myofibroblasts (Cucoranu et al., 2005). Collectively, NOX-mediated oxidative stress plays a significant role in the progression of myocardial fibrosis.

### Molecular signaling pathways involved in cardiac fibrosis

#### TGF-β signaling pathway

Three isoforms of TGF-β (1, 2, and 3) exist in mammals, which are generated by fibroblasts, leukocytes and platelets. TGF-β is primarily produced as a secreted potent form, which is proteolytically activated in a pattern implicated in an integrin-mediated ECM contraction (Sarrazy et al., 2014). The type I TGF-β receptor, also termed activin receptor-like kinase (ALK) 5, predominantly participates in the fibrotic activities of TGF-β. The established pathway of TGF-β1 involved in the activation of ALK5 subsequently phosphorylates Smad2/3; phosphorylated Smad2/3 combines with Smad4 and translocates into the nucleus, where it finally induces the activation of enormous fibrotic genes. TGF-β1 stimulates the collagen lattice contraction and the α-SMA expression in a smad3-dependent fashion (Dobaczewski et al., 2010) Importantly, TGF-β promotes the phenotype conversion of CFs to myofibroblasts and activates ECM component genes to encode fibrillar collagen (Dobaczewski et al., 2011). To inhibit the detrimental effect of TGF-β1 in cardiac remodeling, the ALK inhibitor SM16 is utilized to blunt the TGF-β-induced collagen Iα2 and the lysyl oxidase expression *in vitro*, where it mitigates the cardiac fibrosis in the model of pressure overload (Engebretsen et al., 2014). Moreover, neutralizing anti-TGF-β antibodies decreases the collagen mRNA expression and inhibits fibroblast activation in a rat model of pressure overload (Kuwahara et al., 2002). In addition to the canonical pathway, TGF-β also activates a non-canonical signaling pathway that is implicated in several downstream MAPKs, including c-Jun N-terminal kinase (JNK), P38, and TGF-β-activated kinase 1 (TAK1) (Yan et al., 2009). Each of these MAPKs phosphorylates numerous transcription factors mediate the expression of α-SMA, ECM proteins, and other target genes implicated in cardiac fibrosis. Collectively, the data indicate that the blocking of the downstream TGF-β signaling pathway could be one viable option for anti-fibrotic therapy.

#### Renin–angiotensin–aldosterone system (RAAS)

Comprehensive studies indicate that RAAS activation is consistently detected in fibrotic hearts regardless of etiology. Angiotensin II (Ang II) plays a predominant role in the development of cardiac fibrosis (Kong et al., 2014). *In vitro*, Ang II enhances fibroblast proliferation and migration and induces ECM protein synthesis through the activation of the Ang II type I receptor (AT1R) (Sadoshima and Izumo, 1993; Chen X. Q. et al., 2015). In contrast, AT2R could retard fibroblast proliferation and matrix deposition and work as a negative mediator of the Ang II-induced fibrotic response (Ma et al., 2012). It is also reported that Ang II participates in the TGF-β signaling pathway in CFs (Schultz Jel et al., 2002). Furthermore, activation of AT1R stimulated by Ang II promotes the expression of TGF-β1 and suggests that Ang II enhances the pathological role of TGF-β1 in inducing cardiac hypertrophy and fibrosis (Schultz Jel et al., 2002). Moreover, collagen expression of fibroblasts induced by AngII requires the activation of the TGF-β/Smad and MAPK signaling pathway. Collectively, Ang II signaling can induce myofibroblast differentiation through enhancement of TGF-β1 expression and the activation of canonical and non-canonical signaling effectors, and/or directly activating MAPK-serum response factor (SRF) signaling (Davis and Molkentin, 2014).

#### Connective tissue growth factor (CTGF)

CTGF, also known as CNN2, is significantly upregulated in human heart failure and the myocardial fibrosis associated animal model (Chuva de Sousa Lopes et al., 2004; Koshman et al., 2013). Factors including TGF-β, Ang II, ET-1, and mechanical stress can induce the expression of CTGF in cardiomyocytes and fibroblasts (Travers et al., 2016). Intriguingly, the expression of CTGF is increased before the upregulation of TGF-β, suggesting an important role in cardiac fibrosis. Surprisingly, there is neither a gain nor a loss of CTGF in the cardiac tissue influencing cardiac function and fibrogenesis. Moreover, mediation of CTGF slightly affects the response to TGF-β in pressure-overload-induced heart failure. The data above suggest that CTGF is less important as a TGF-β effector and to elucidate the potential role in cardiac fibrosis.

#### Platelet-derived growth factor (PDGF)

PDGF has been involved in the progression of cardiac fibrosis by inducing the synthesis of TGF-β. However, TGF-β could also enhance the production of PDGF (Czuwara-Ladykowska et al., 2001; Zhao et al., 2013). Following MI, PDGF contributes to collagen accumulation, fibroblast proliferation, and enhances scar reparation (Al Hattab and Czubryt, 2017). Cardiac-specific overexpression of PDGF markedly increases the expression of TGF-β1 and contributes to the development of cardiac fibrosis (Tuuminen et al., 2009). These results indicate that PDGF plays a potential role in cardiac fibrosis and appears to be suggesting a potential target for anti-fibrotic therapy.

## Anti-fibrotic effect of polyphenols on cardiac fibrosis

Polyphenols contain several phytochemicals sharing a common phenolic structure, and they are divided into flavonoids and non-flavonoids (Del Rio et al., 2013). Flavonoids are formed by 15 carbons with two aromatic rings connected by a three-carbon bridge, indicated as C6-C3-C6. The main subclasses of flavonoids are divided into flavones, isoflavones, flavonols, flavan-3-ols, flavanones, and anthocyanidins. Non-flavoniods do not possess the C6-C3-C6 structure and could formed by a single phenolic ring combined to one or three carbons such in phenolic acids or hydroxycinnamic acids; another representative non-flavonoids is still benes with a C6-C2-C6 stricture such as resveratrol (Salomone et al., 2016). Some polyphenols are characterized by the inhibition of cardiac fibrosis (Jiang et al., 2010). In this section, we aimed to summarize the existing experimental evidence regarding polyphenols and cardiac fibrosis (Table 1).

**Table 1:** Anti-fibrotic effects of polyphenols on cardiac fibrosis.

**Study type**	**Class**	**Polyphenols**	**Study design**	**Dose**	**Actions**	**Reference**
*In vivo*	Flavonols/flavan-3-ols	Quercetin	Male Wistar rats induced by ISO	25 or 50 mg/kg	↓TGF-β1, CTGF ↓Deposition of ECM	Li et al., 2013
		Taxifolin	Male C57/BL6 mice induced by TAC	Feed containing 0.2% taxifolin	↓TGF-β, Collagen I, Collagen III, CTGF ↓TGF-β/Smad signaling pathway	Guo et al., 2015
		Isorhamnetin	C57/BL6 mice induced by TAC	100 mg/kg/day for	↓Collagen I, Collagen III, CTGF ↓PI3K–AKT signaling pathway	Gao et al., 2017
		Troxerutin	Mus musculus fed HFFD	150 mg/kg	↓TGF-β, α-SMA, NOX p22phox ↓MMP-9/TIMP-1 and MMP-2/TIMP-2 ratio	Geetha et al., 2015
		(-)-epicatechin	δ-sarcoglycan (δ-SG) null mice	1 mg/kg	↑SOD2, catalase, and citrate synthase ↓Cardiac fibrosis	Ramirez-Sanchez et al., 2014
		Catechins	Lewis rats immunized with PCM	20 mg/kg/day	↓T cell proliferation ↓TNF-α, IL-2, cardiac fibrosis	Suzuki et al., 2007
	Flavones/isoflavones	Luteolin	SD rats induced by Ang II	2.3 g and 3.5 g/10 kg chow	↓NOX2 and NOX4 ↓P-JNK and TGF-β1	Nakayama et al., 2015
		L7DG	C57BL/6J mice induced by ISO	40 mg/kg	↓NOX subunits: Cyba, Cybb, Ncf1, Ncf4, Rac2 ↓Col1a1, Col1a2, Col3a1, Col12a1, Fbn1	Ning et al., 2017
		Baicalein/	SHR	50, 200 mg/kg	↓P-ERK and MMP-9	Kong et al., 2011
		Apigenin	Wistar rats induced by AMI	10, 20, and 40 mg/kg	↓TNF-α, IL-1β, IL-6 ↓MMP-9 and NF-κB	Du et al., 2015
		Scutellarin	Wistar rats induced by LAD ligation	10 and 30 mg/kg	↓TGF-β1 and FN1 expression ↓P38-MAPK and ERK1/2	Pan et al., 2011
		Scutellarin	SD rats induced by ISO	10, 20 mg/kg	↓α-SMA ↑CD31, Jagged1, Notch 1, and Hes1	Zhou et al., 2014
		Genistein	Kunming mice induced by TAC	50 and 100 mg/kg/day	↓Myofibroblast transformation ↓TAK1/MKK4/JNK signaling	Qin et al., 2015
	Anthocyanins	Cy3G	Wistar rats induced by STZ	250mg/kg/day	↑TIMP-1 ↓MMP-9,.TGF-β, p-MEK1/2, CTGF, P-ERK1/2, FGF2	Chen et al., 2016
	Flavanones	Hesperetin	C57 mice subjected to AB	30 mg/kg/day	↓PKCα/βII/AKT and JNK activation ↓TGF-β1/Smad signaling	Deng et al., 2013
	Non-flavoniods	Resveratrol	C57/BL6 mice induced by TAC	10 mg/kg	↓Macrophage and mast cell infiltration ↓Oxidative stress, cardiac fibrosis	Gupta et al., 2014
		Resveratrol	SD rats subjected to AAC	4 mg/kg	↑SERCA2 expression ↓P-CaMKII and cardiac fibrosis	Dong et al., 2014
		Resveratrol	C57Bl/6 mice subjected to TAC	4 g/kg AIN-93G diet	↓Collagen 1α1 and collage 3α1, MMP-2, TIMP-1/2/3/4, collagen deposition	Sung et al., 2015
		Resveratrol	C57Bl/6 mice induced by STZ	5 or 25 mg/kg	↓ROS/ERK/TGF-β/periostin pathway	Wu et al., 2016
		Resveratrol	Fischer 344 rats induced by DOX	2.5 mg/kg	↑SIRT-1 expression ↓TGF-β/Smad3 pathway	Cappetta et al., 2016
		Resveratrol	Wistar rats induced by DOX	20 mg/kg/day	↓Caspase-3, TNF-α, MDA, and TGF-β1 ↓Collagen accumulation	Arafa et al., 2014
		Resveratrol	DOCA-salt induced hypertensive rats	1 mg/kg/day	↓left ventricular collagen content	Chan et al., 2011
	Other	Curcumin	SD rats subjected to I/R	150 mg/kg	↓MMP-9, MMP-2, collagens synthesis ↓TGF-β/Smad pathway	Wang et al., 2012
		Curcumin	SD rats subjected to I/R	300 mg/kg	↓Toll-like receptor 2 and MCP-1 ↓Macrophage infiltration, cardiac fibrosis	Kim et al., 2012
		Curcumin	C57BL/6 mice fed HFD;	50 mg/kg	↑Nrf-2 expression ↓NF-κB, TNF-α, IL-1β, and IL-6, cardiac fibrosis	Zeng et al., 2015
		Curcumin	HFD fed wistar rats induced by STZ	200 mg/kg	↓gp91phox and p47phox ↑P-Akt, P-GSK-3β	Yu et al., 2012
		Curcumin	SD rats induced by STZ	300 mg/kg	↓collagen types I and III synthesis ↓TGF-β1/Smad signaling	Guo et al., 2017
		C66	C57BL/6 mice induced by STZ	5 mg/kg	↓P-JNK expression ↓TNF-α, IL-6, collagen IV, TGF-β	Pan et al., 2014
		C66	C57BL/6 mice induced by STZ	5 mg/kg every other day	↓P-JNK, CTGF, TGF-β1	Wang et al., 2014
		Piperine	C57BL/6 mice induced by AB	50 mg/kg *in vivo*	↓AKT/GSK3β signaling ↑PPAR-γ	Ma Z. G. et al., 2017
		Evodiamine	C57BL/6 mice induced by ISO	50 and 100mg/kg/day	↓Endothelial-to-mesenchymal transition ↓TGF-β/Smad pathway	Jiang et al., 2017
*In vitro*	Flavonols/flavan-3-ols	EGCG	CFs s induced by Ang II	12.5-200 mg/L 10 umol/L 10 umol/L	↓CFs proliferation ↓CFs proliferation ↓CFs proliferation ↓CTGF, NF-κB signaling pathway	Sheng et al., 2009; Cai et al., 2013; Han et al., 2013
				100 μmol/L	↓P-JNK and endoglin expression ↓CFs proliferation	Lin et al., 2016
	Flavones/isoflavones	Luteolin	CFs stimulated by Ang II	6.2, 12.5, and 25 μmol/L	↓CFs proliferation and collagen synthesis ↑NO-cGMP signal pathway	Wang A. W. et al., 2015b
		Baicalein/Wogonin	CFs stimulated by Ang II	30 μmol/L; 30 μmol/L	↓Collagen I, III expression ↓Collagen I, III expression	Kong et al., 2010
	Non-flavoniods	Resveratrol	CFs stimulated by AngII	80μmol/L	↓TGF-β1/smad3 pathway ↓CFs proliferation, myofibroblast transformation	Chen T. et al., 2015
	Other	Curcumin	CFs stimulated by Ang II	10 and 15 μmol/L	↓CFs proliferation and migration, MMP-2/9 ↑SIRT-1 expression	Xiao et al., 2016
		Curcumin	CFs stimulated by Ang II	5,10, and 20 umol/L	↓CFs proliferation ↓TGF-β1/Smad2/3 pathway	Meng et al., 2014
		Curcumin	CFs induced by Ang II	10 and 20 μmol/L *in vitro*	↓TGF-β1, MMP-9, TIMP-1, α-SMA ↓Fibroblast proliferation	Ma J. et al., 2017
		Curcumin	CFs induced by TGF-β1	5, 10, and 20 μmol/L	↓α-SMA and Col I ↓P-Smad2 and P-P38	Liu et al., 2016
		Curcumin	H9C2 cells induced by PA	20 μmol/L	↓TGF-β	Zeng et al., 2015
		Evodiamine	CFs and HUVECs stimulated by TGF-β1	5, 10 μmol/L	↓α-SMA, collagen I and III, fibronectin„ CTGF ↑CD31, CD34 expression ↓P-Smad2, P-Smad3, P-ERK1/2, P-Akt	Wu et al., 2017a

*ISO, isoproterenol; HFD, high-fat diet; HCHF, high carbohydrate, high fat diet; TAC, thoracic aorta constriction; STZ, streptozotocin; AAC, abdominal aortic constriction; SD, Sprague-Dawley; CFs, Cardiac fibroblasts; AMI, acute myocardial infarction; NOX, NAPDH oxidase; SUMO1, small ubiquitin-related modifier 1; MI, myocardial infarction; LAD, left anterior descending coronary artery; SHR, spontaneously hypertensive rats; MMP, matrix metalloprotease-9; AB, aortic banding; COX-2, cyclooxygenase-2; ECFC, endothelial colony-forming cell; TIMP-1, tissue inhibitor of matrix metalloproteinase-1; CaMK II, Ca^2+^ /calmodulin-dependent protein kinase II; DOCA, deoxycorticosterone acetate; I/R, ischaemia reperfusion; NRCMs, neonatal rat cardiomyocytes; ROS, reactive oxygen species; L7DG, luteolin-7-diglucuronide; FN, fibronectin; Cy3G, cyanidin-3-glucoside; FGF, fibroblast growth factor; ECM, extracellular matrix ; HFFD, high-fat high-fructose diet ; DOX, doxorubicin; SOD, Superoxide dismutase; PCM, porcine cardiac myosin*.

### Flavonols and flavan-3-ols in cardiac fibrosis

Quercetin, one of the most widely distributed flavonols, is abundant in red onions, citrus fruits, grains, and many other foods of plant origin (Pawlikowska-Pawlega et al., 2003). The quercetin found in dietary bioflavonoids coexists with its glycoside derivative, rutin. Studies have demonstrated that rutin was more soluble than quercetin. In the animal model of cardiac fibrosis, the administration of quercetin and rutin or single quercetin attenuated cardiac dysfunction and myocardial injury stimulated by isoproterenol (ISO) and prevented cardiac fibrosis by inhibition of CTGF, TGF-β1, and ECM deposition (Li et al., 2013). Panchal et al. indicated that in a model of an obese rat being fed a western diet supplemented with quercetin, cardiac remodeling was prevented via the inhibition of the NF-κB signaling pathway and the promotion of the nuclear factor erythroid 2-related factor 2 (Nrf-2) and its downstream molecules (Panchal et al., 2012). Taxifolin, one of quercetin derivatives, has the ability to blunt cardiac fibrosis induced by pressure overload, the mechanism underlying anti-fibrotic effect of taxifolin dependents on inhibition of TGF-β/Smad signaling pathway (Guo et al., 2015). Isorhamnetin, another ingredient of flavonols, ameliorates cardiac hypertrophy and fibrosis induced by aortic banding (Gao et al., 2017). Consistent with the results above, *Boerhavia diffusa* extracted with ethanol (BDE) is a rich source of bioactive flavonols containing quercetin, boeravinone, kaempferol, and caffeic acid, mitigating Ang II-induced cardiac fibrosis via the inhibition of TGF-β1 expression and collagen deposition (Prathapan et al., 2017). In recent years, the metabolic effect of rutin and troxerutin have been examined in robust animal models of metabolic syndrome. Anuradha et al. demonstrated that troxerutin enhances insulin sensitivity, reduces lipid accumulation and upregulates fatty acid oxidation in the heart (Geetha et al., 2014); furthermore, troxerutin slowed the fibrotic response in the myocardium following the long-term feeding of a high-fat high-fructose diet (Geetha et al., 2015).

The beneficial effect of green tea has been attributed to the presence of abundant catechins. Epigallocatechin-3-gallate (EGCG) is the most abundant and powerful catechin in green tea (Mak, 2012). EGCG inhibits CFs proliferation in rats and alleviates cardiac hypertrophy (Sheng et al., 2009). Guo et al. indicated that EGCG retarded cardiac hypertrophy *in vivo* and *in vitro* via the inhibition of oxidative stress (Sheng et al., 2007). Meanwhile, it attenuated the activation of rat CFs stimulated by AngII via the mediation of β-arrestin1 (Han et al., 2013). Liu et al. found that EGCG could decrease collagen synthesis and fibronectin expression in rat CFs induced by Ang II; moreover, it markedly ameliorated the excessive expression of CTGF and cardiac fibrosis via the blockage of the NF-κB signaling pathway in the hypertrophic stimulation (Cai et al., 2013). Additionally, EGCG inhibits the expression of endoglin stimulated by Ang II in CFs via the blockage of the JNK signaling pathway, thus slowing down the CFs proliferation *in vitro* and mitigating reparative scar fibrosis following MI (Lin et al., 2016). In the model of muscular dystrophies, epicatechin attenuates oxidative stress and improves mitochondrial function, thus decreasing heart fibrosis in δ-sarcoglycan null mice (Ramirez-Sanchez et al., 2014). Catechin administration decreases tumor necrosis factor (TNF-α) and Th2 cytokines secretion in the heart tissues and mitigates cardiac fibrosis in rat autoimmune myocarditis (Suzuki et al., 2007). However, the protective role of EGCG in attenuating cardiac fibrosis depends on a proper dose. Conversely, a high dose of EGCG results in cardiac collagen synthesis and aggravates cardiac fibrosis in mice (Cai et al., 2015).

### Flavones/isoflavones in cardiac fibrosis

Luteolin is one of the flavones that can be extracted from thyme, onion, broccoli, and cauliflower. It inhibits CFs proliferation via the reduction of oxidative stress *in vitro* (Wang T. et al., 2015), the mechanism where underlying anti-oxidative stress depends on the inhibition of NOX2 and NOX4 in cardiac hypertrophy, thus decreasing the phosphorylation of JNK and TGF-β1 expression and alleviating cardiac fibrosis (Nakayama et al., 2015). Zhang et al. have indicated that luteolin-7-diglucuronide, another flavonoid glycoside, prevents ISO-induced myocardial fibrosis resulting from the downregulation of NOX and fibrogenesis-associated gene expression (Ning et al., 2017). Baicalein and wogonin are two of the main active ingredients in the *Scutellaria baicalensis* Georgi. In the CFs, baicalein and wogonin treatment suppresses collagen I and collagen III expression stimulated by Ang II (Kong et al., 2010). They also reduce myocardial collagen volume fraction and inhibit myocardial collagen I and III mRNA expression in spontaneously hypertensive rats by mediating ERK and MMP-9 pathways (Kong et al., 2010, 2011). Our lab indicated that baicalein treatment alleviated cardiac hypertrophy *in vivo* and *in vitro*. The mechanism underlying an anti-hypertrophic response resulted from the inhibition of MAPK kinase (MEK)-ERK1/2 signaling and GATA-4 activation, and alleviation of interstitial fibrosis was observed in hypertrophic cardiac tissues following the administration of baicalein (Zong et al., 2013). In another cardiac hypertrophy model stimulated by Ang II infusion, baicalein also reduced Ang II-induced myocardial hypertrophy and collagen deposition (Wang A. W. et al., 2015). Consistent with previous studies about luteolin, baicalein not only attenuates cardiac fibrosis but also prevents the downregulation of SERCA2a and modulates intracellular Ca^2+^ concentration (Zhao et al., 2016). Apigenin, one of the flavones, modulates the activity of PPAR-γ and the glucose/lipid metabolism (Maron, 2004). Recent studies indicate that apigenin also attenuates myocardial injury induced by ISO via regulating the activity of PPAR-γ in diabetic rats (Buwa et al., 2016). Our group demonstrated that the administration of apigenin mitigated cardiac remodeling via inhibition of oxidative stress, the NF-κB pathway and apoptosis, and reduced cardiac interstitial fibrosis in STZ-induced diabetic cardiomyopathy (Liu et al., 2017). Furthermore, due to anti-inflammatory and anti-oxidative properties, apigenin treatment inhibits matrix metalloprotease-9 and inflammatory reactions after acute myocardial injury (Du et al., 2015; Gutiérrez-Venegas and González-Rosas, 2017) and decreases cardiac fibrosis in the progression of MI (Gutiérrez-Venegas and González-Rosas, 2017). In a rat cardiac hypertrophy model induced by renovascular hypertension, administration of apigenin improved hypertensive cardiac dysfunction and abnormal myocardial glucolipid metabolism (Zhu et al., 2016). Similarly, scutellarin, as one of the members of the flavones, inhibits the proliferation and collagen production of CFs *in vitro* and suppresses the up-regulation of fibronectin and TGF-β1 induced by Ang II (Pan et al., 2011). Recent studies elucidate that the anti-fibrotic effect of scutellarin could be attributed to the inhibition of endothelial–mesenchymal transition (EndoMT) following the stimulation of ISO in a rat (Zhou et al., 2014). Other flavones such as nobiletin (Parkar et al., 2016; Zhang et al., 2016), vitexin (Dong et al., 2011; Che et al., 2016), tangerentin (Vaiyapuri et al., 2013) not only possess an anti-oxidative and anti-inflammatory therapeutic effect but also exert a potent anti-fibrotic effect in an experimental animal model of cardiovascular diseases.

Isoflavones, such as genistein and daidzein, are present in large quantities in soybeans and exert beneficial anti-fibrotic effects on cardiac remodeling. Recent studies indicate that a genistein supplement attenuates ISO-induced cardiac hypertrophy in rats (Maulik et al., 2012) and inhibits TGF-β1-induced proliferation, collagen production and myofibroblast transformation (Qin et al., 2015). Genistein treatment enhances endothelial colony-forming cell (ECFC) proliferation and migration, and transplants of genistein-stimulated ECFCs into myocardial ischemic sites *in vivo* stimulate cell proliferation and secretion of angiogenic cytokines at the ischemic sites, thereby alleviating myocardial fibrosis after cardiac function (Lee et al., 2014).

### Anthocyanins in cardiac fibrosis

Anthocyanins such as malvidin-3-glucoside, delphinidin-3-glucoside (Dp3G), cyanidin-3-glucoside (Cy3G), petunidin-3-glucoside (Pg3G), and peonidin-3-glucoside from grape skins exert protective effects over the complication of ischemia/reperfusion (Liobikas et al., 2016) and diabetes mellitus (Sun et al., 2012). Dp3G and Cy3G, but not Pg3G, could recover ischemia-induced damage of complex I of the mitochondrial respiratory chain and increase ischemia-depleted ATP levels by promoting oxidative phosphorylation (Skemiene et al., 2015). Due to the high capacity to decrease cytosolic cytochrome c, Cy3G, but not Pg3G, prevents the rat heart from ischemia/reperfusion-induced apoptosis and necrosis (Škemiene et al., 2013). Administration of Cy3G attenuates cardiac dysfunction and cardiac inflammation in STZ-induced diabetic cardiomyopathy and decreases collagen disposition via activation of matrix metalloproteinase-9 (MMP-9) and reduction in the level of tissue inhibitor of matrix metalloproteinase (TIMP)-1 observed in diabetic rat heart (Chen et al., 2016). When taken together, experimental data suggest that anthocyanins with high reductive capacity can decrease cytosolic cytochrome c, inhibit caspase-associated apoptosis and necrosis in the ischemic/reperfused myocardium, and rescue deteriorating fibrosis in a diabetic rat heart. However, the anti-oxidative and anti-fibrotic effect on other cardiovascular diseases, such as MI and pressure overload, still need to be well-investigated.

### Flavanones in cardiac fibrosis

The flavanone hesperitin, presented in citrus peels, has been shown to possess beneficial cardiovascular effects in different animal models (Roohbakhsh et al., 2015). The increased intake of flavanones tends to reduce the incidence of coronary heart diseases (Hertog et al., 1993; Geleijnse et al., 2002). Due to its anti-oxidative and anti-apoptosis properties, hesperitin reversed doxorubicin (Dox)-induced oxidative stress and decreased apoptosis in H9C2 cells stimulated by lipopolysaccharide through the mitochondria-dependent intrinsic apoptotic pathway (Yang et al., 2014). The inhibitory effect of hesperitin on cardiac remodeling by blocking the JNK and TGFβ1/Smad signaling pathways and mitigating fibrosis has been reported by our research group (Deng et al., 2013). Hesperidin, as a flavanone glycoside, plays a protective role in ISO-induced myocardial ischemia through the inhibition of lipid peroxidative and oxidative stress (Selvaraj and Pugalendi, 2010). Hesperidin could also increase the mRNA expression of Nrf-2 to exert a protective role in the heart of aged rats (Elavarasan et al., 2012). Moreover, a significant decrease in cardiac function biomarkers, including serum creatine kinase, aspartate aminotransferase, and lactate dehydrogenase, in diabetic rats has been detected following hesperidin supplement (Mahmoud et al., 2012).

Another citrus flavanone, naringenin, has been shown to possess protective effects on lipid metabolism. Mulvihill elegantly indicated that naringenin treatment ameliorated dyslipidemia, reduced increased VLDL levels and improved insulin sensitivity in Ldlr^−/−^ mice fed a western diet through a PPAR-γ coactivator 1α/PPAR-α mediated transcription program (Mulvihill et al., 2009). In a H_2_O_2_-treated cardiomyoblast, naringenin treatment attenuated stress-induced apoptotic cell death and lipid peroxidation and increased the level of reduced glutathione, whose protective effect mainly depends on the upregulation of Nrf-2 and downregulation of the NF-κB signaling pathway (Ramprasath et al., 2014). Due to its significantly anti-oxidative effect, recent studies indicate that naringenin plays a beneficial role in the models of age-associated cardiac disorders (Da Pozzo et al., 2017), hyperglycemia-induced cardiomyocyte injuries (Chtourou et al., 2015; You et al., 2016) and Dox-induced rat cardiotoxicity (Subburaman et al., 2014). Our results reveal that administration of naringenin ameliorates cardiac hypertrophy by inhibiting the activation of JNK, ERK, and phosphatidylinositol-3-kinase/Akt signaling pathways, and it mitigates myocardial fibrosis (Zhang et al., 2015); however, the mechanism underlying the anti-fibrotic effect needs to be clarified.

### Some non-flavonoids in cardiac fibrosis

Resveratrol is a naturally occurring polyphenol mainly contained in plants. A supplement of resveratrol has been shown to prevent and/or slow down the progression of cardiac remodeling in multiple animal models of heart failure (Sung and Dyck, 2015). Recent studies have shown that treatment of resveratrol could activate sirtuins-3 (SIRT-3) and decrease collagen accumulation. Moreover, *in vitro* studies indicated that resveratrol treatment inhibited CFs proliferation and fibroblast-to-myofibroblast transition stimulated by Ang II via blunting the TGF-β/Smad3 pathway (Chen T. et al., 2015). Resveratrol can activate adenosine 5-monophosphate-activated protein kinase (AMPK) and extend the rat's lifespan, while it loses its protective effect in AMPKα1/α2-knockout mice (Um et al., 2010). Treatment of resveratrol reversed cardiac remodeling and mitigated cardiac dysfunction by enhancing autophagy via the activation of AMPK (Kanamori et al., 2013). Ren et al. demonstrated that treatment with resveratrol mitigated aging-induced ${\text{O}}_{2}^{-}$ generation and mechanical dysfunction in cardiomyocytes stimulated by aldehyde dehydrogenase 2 activator Alda-1 (Zhang et al., 2014). Resveratrol supplement has been shown to suppress the interstitial and perivascular fibrosis induced by pressure overload in mice and rats (Dong et al., 2014; Gupta et al., 2014). Dyck et al. demonstrated that the mRNA expression of collagen I and III, MMP-2 TIMP-1/2/3/4, and collagen deposition was suppressed by resveratrol, and its anti-fibrotic effect was partially attributed to AMPK activation (Sung et al., 2015). Meanwhile, resveratrol is known for its anti-oxidant and anti-inflammatory properties (Bonnefont-Rousselot, 2016). Supplementation of resveratrol exhibited an anti-proliferative effect on CFs via blocking the ROS/ERK pathway and attenuated fibroblasts-myofibroblast transition via inhibiting the ROS/ERK/TGF-β/periostin pathway in STZ-induced diabetic cardiomyopathy (Wu et al., 2016). In dox-induced cardiotoxicity, it has been found that fibroblasts isolated from the resveratrol-treated group have decreased levels of TGF-β/Smad3 expression and up-regulated sirt1 expression; consistent with the results *in vivo*, resveratrol supplement mitigates myocardial stiffness and collagen deposition (Cappetta et al., 2016). It was shown that Dox up-regulated not only TGF-β1 expression but also stimulated massive collagen accumulation in left ventricle tissues, whereas the dox-induced fibrotic effect was attenuated by resveratrol (Arafa et al., 2014). In another study, the anti-fibrotic effect on cardiac remodeling was observed in deoxycorticosterone acetate-treated rats following resveratrol treatment (Chan et al., 2011). Although the studies about the anti-fibrotic effect of resveratrol are very scarce, preclinical studies have obtained promising and, in some cases, exciting results for the retardation and/or therapy of hypertension and ischemia-reperfusion injuries (Zordoky et al., 2015). Therefore, if these exciting animal results translate to humans, resveratrol may provide a novel and promising agent for the treatment of cardiovascular diseases.

### Curcumin

Previous studies have shown that curcumin, a natural p300-specific histone acetyltransferase (HAT) inhibitor, exerts a therapeutic effect on heart failure (Sunagawa et al., 2011). In the MI, curcumin only or combined with enalapril improves the left ventricular systolic function by inhibiting nuclear expression of p300 and mitigates extensive perivascular fibrosis in rats (Sunagawa et al., 2011, 2014). Another study has shown that curcumin mitigates maladaptive cardiac repair and reduces cardiac fibrotic response after ischemia and reperfusion by decreasing ECM degradation and inhibiting the synthesis of collagens via the TGF-β/Smad pathway (Wang et al., 2012). Another study revealed that curcumin significantly ameliorated collagen accumulation *in vivo* and inhibited CFs proliferation and migration as well as MMP expression. Moreover, curcumin pretreatment downregulated the expression of SIRT-1 after MI, which demonstrated that the activation of SIRT1 was implicated in the beneficial role of curcumin (Xiao et al., 2016). In spontaneously hypertensive rats, treatment with curcumin decreases the expression of fibrotic markers CTGF, collagen III, and fibronectin. The inhibitory effect of collagen synthesis in the isolated fibroblasts stimulated by Ang II depends on inhibiting the TGF-β1/Smad2/3 pathway (Meng et al., 2014). Moreover, curcumin can also inhibit fibroblast differentiation in ISO and TGF-β stimulated CFs (Liu et al., 2016; Ma et al., 2016) and ameliorate myocardial collagen deposition. Furthermore, curcumin treatment suppresses the increased expression of toll-like receptor 2 and MCP-1 in cardiomyocytes following TNF-α stimulation or ischemia-reperfusion injury and decreases the fibrotic response observed in cardiac tissues (Kim et al., 2012). The protective role of curcumin is closely associated with its capacity to up-regulate Nrf-2 expression and repress NF-κB activation (Zeng et al., 2015). Curcumin could regulate the Akt/GSK-3β signaling pathway and NOX expression in diabetic cardiomyopathy and ameliorate cardiac fibrosis(Yu et al., 2012); t the anti-fibrotic mechanism is associated with the inhibition of collagen type I and III synthesis and TGF-β1/Smad signaling (Guo et al., 2017). Additionally, the same cardioprotective effect is achieved by a new mono-carbonyl curcumin analog, Y20 (Qian et al., 2015). A novel curcumin derivative, C66, has ability to mitigate high glucose-induced inflammatory and apoptosis and inhibit JNK phosphorylation in both H9C2 cells and neonatal cardiomyocytes, and C66 treatment also improves cardiac function and extensive fibrosis in diabetic mice In addition, the same cardioprotective effect was achieved by new mono-carbonyl curcumin analog, Y20(Pan et al., 2014). Moreover, cardiac metallothionein expression and fibrosis is markedly suppressed by administration of C66 via inhibiting the diabetic upregulation of JNK phosphorylation in mice (Wang et al., 2014).

### Piperine/evodiamine

A previous study found that piperine could attenuate weight gain in rodents (BrahmaNaidu et al., 2014), and the beneficial effect of piperine was associated with the up-regulation of the metabolic rate of resting muscle (Nogara et al., 2016). In the metabolic syndrome model, piperine reduced ventricular wall thickness and attenuated cardiac collagen deposition in rats fed a high-fat diet (Diwan et al., 2013). Furthermore, pretreatment of piperine ameliorates oxidative stress, dyslipidemia, and fibrosis in ISO-induced myocardial ischemia (Dhivya et al., 2017). Our lab has reported that piperine administration mitigates cardiac fibrosis induced by pressure-overload or ISO simulation in mice. Isolated CFs transition induced by TGF-β could be attenuated by piperine via inhibition of the AKT/GSK3β pathway (Ma Z. G. et al., 2017). Endothelial-to-mesenchymal transition participates in cardiac fibrosis in the progression of cardiac remodeling. Evodiamine is the major ingredient isolated from the fruit of *E. rutaecarpa*. Our study indicated that evodiamine ameliorated cardiac dysfunction and retarded cardiac fibrosis induced by ISO stimulation via mediating EndoMT *in vivo* (Jiang et al., 2017); the anti-fibrotic effect and mechanism in fibroblast stimulated by TGF-β was observed following evodiamine administration (Wu et al., 2017a).

## Challenges and future direction

**Figure 2:**
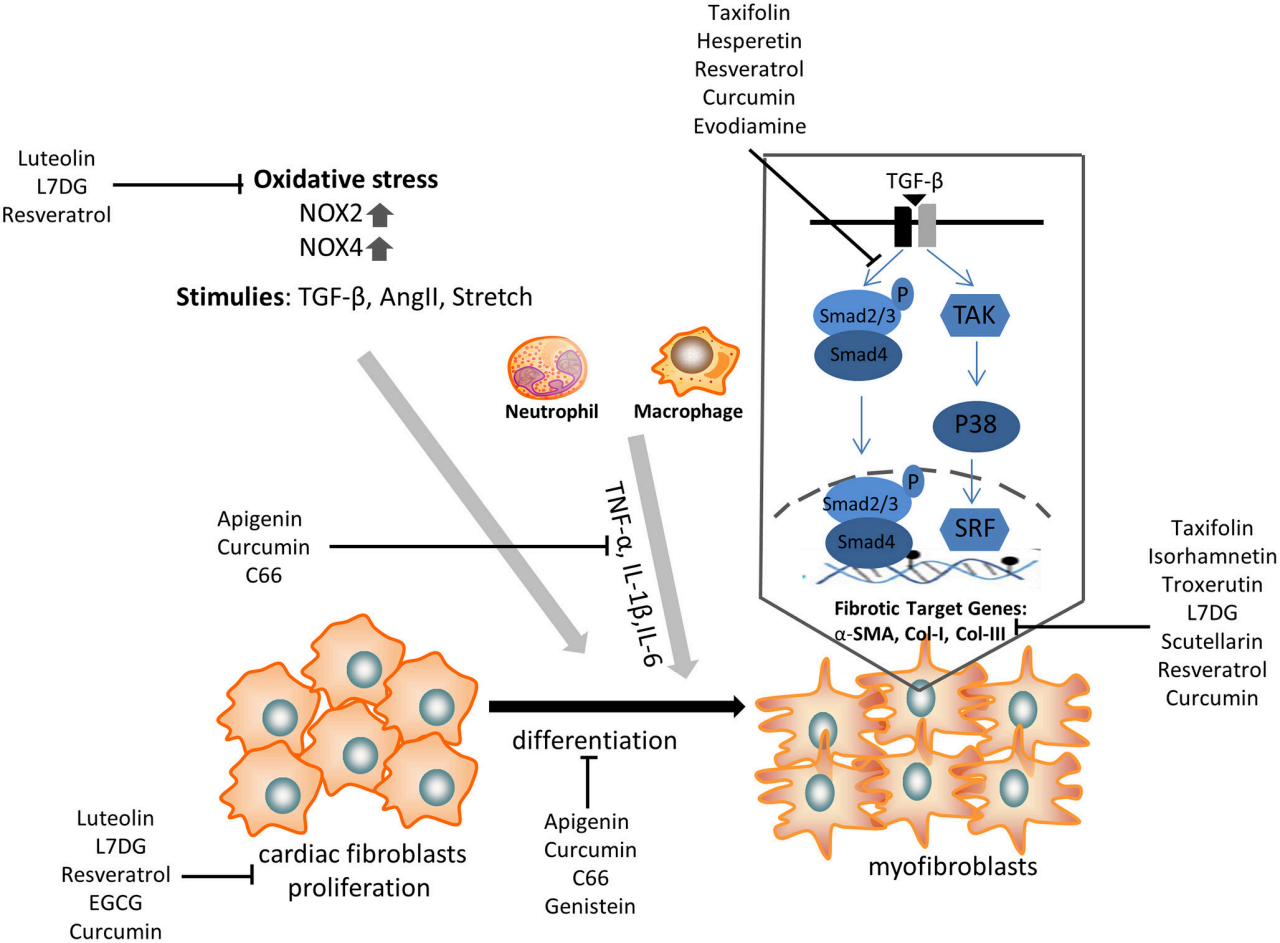
The anti-fibrotic effect of some selected polyphenols on cardiac fibroblasts proliferation, transdifferentiation, extracellular matrix deposition, and TGF-β/smad signaling pathway. α-SMA, α-smooth muscle actin; NOX, NAPDH oxidase; TNF, tumor necrosis factor; IL, interleukin; TGF, transforming growth factor; L7DG, luteolin-7-diglucuronide.

Cardiac fibrosis is a prevalent pathology in response to stress and injury. It is abundantly clear that the extensive understanding of fibrosis-associated mechanisms is critical to obtain exciting advancements in the treatment of cardiac fibrosis. Given the complicated development of cardiac fibrosis, conventional therapies, such as β-blockers and RAAS inhibitors, do not completely hamper the progression of cardiac fibrosis in patients with heart failure. Therefore, an additional or novel treatment with some polyphenols might provide a potential strategy to alleviate cardiac fibrosis, as it has been shown to target CFs differentiation and pro-fibrotic molecular signals (Figure 2).

Many epidemiological studies have indicated that the consumption of natural polyphenols is associated with a reduced risk of suffering from chronic diseases. The increased intake of fruits and vegetables is inversely associated with major cardiovascular diseases, MI, and cardiovascular mortality (Miller et al., 2017). A dietary supplement of polyphenols provides a convenient way to prevent the occurrence of cardiovascular disease and fibrosis. Moreover, some polyphenols possess anti-oxidant and anti-inflammatory properties, and comprehensive animal experimental studies found that some polyphenols could be regarded as a potential agent to treat cardiac fibrosis. Multiple lines of evidence exploring the cardioprotective effects of resveratrol have obtained promising results; however, there is still a long way to determine whether resveratrol will finally translate from the laboratory to the clinic (Rauf et al., 2017). Despite some progress made in polyphenols research in animal models of cardiac fibrosis, there are major gaps that need to be settled. First, long-term trials (animal and human) must be performed to evaluate the therapeutic effect and toxicity of polyphenols. Second, the efficiency and bioavailability of polyphenols in its absorption and metabolism need to be considered. As the dose of polyphenols will have to be examined, consideration must be whether made as to administration with individual flavonoids, flavonoid combinations, or simply dietary recommendations on polyphenols supplement represents the effective approach for the rapid translation of these lab results into effective interventions for cardiac fibrosis. Thus, understanding the anti-fibrotic targets of polyphenols and their structure-activity relationship, improved screening methods, and achieved positive results of clinical trials will be critical to specify the future direction for new drug discovery. Collectively, our review suggests that some polyphenols in experimental studies could target fibrotic mechanisms and thus may be a potential therapeutic agent for cardiac fibrosis.

## Author contributions

NZ and W-YW were responsible for assembling and drafting of the manuscript. L-LL, CH, and Q-ZT contributed to the drafting of the manuscript.

### Conflict of interest statement

The authors declare that the research was conducted in the absence of any commercial or financial relationships that could be construed as a potential conflict of interest.
